# Plant Secondary Metabolites as Anticancer Agents: Successes in Clinical Trials and Therapeutic Application

**DOI:** 10.3390/ijms19010263

**Published:** 2018-01-16

**Authors:** Ana M. L. Seca, Diana C. G. A. Pinto

**Affiliations:** 1 cE3c—Centre for Ecology, Evolution and Environmental Changes/Azorean Biodiversity Group & Faculty of Sciences and Technology, University of Azores, Rua Mãe de Deus, 9501-321 Ponta Delgada, Portugal; 2 Department of Chemistry & QOPNA—Organic Chemistry, Natural Products and Food Stuffs, University of Aveiro, Campus de Santiago, 3810-193 Aveiro, Portugal; diana@ua.pt

**Keywords:** secondary metabolites, clinical trial, anticancer therapy, vincristine, paclitaxel, homoharringtonine, ingenol mebutate, curcumin, betulinic acid

## Abstract

Cancer is a multistage process resulting in an uncontrolled and abrupt division of cells and is one of the leading causes of mortality. The cases reported and the predictions for the near future are unthinkable. Food and Drug Administration data showed that 40% of the approved molecules are natural compounds or inspired by them, from which, 74% are used in anticancer therapy. In fact, natural products are viewed as more biologically friendly, that is less toxic to normal cells. In this review, the most recent and successful cases of secondary metabolites, including alkaloid, diterpene, triterpene and polyphenolic type compounds, with great anticancer potential are discussed. Focusing on the ones that are in clinical trial development or already used in anticancer therapy, therefore successful cases such as paclitaxel and homoharringtonine (in clinical use), curcumin and ingenol mebutate (in clinical trials) will be addressed. Each compound’s natural source, the most important steps in their discovery, their therapeutic targets, as well as the main structural modifications that can improve anticancer properties will be discussed in order to show the role of plants as a source of effective and safe anticancer drugs.

## 1. Introduction

Although cancer is the most devastating disease, causing more deaths than all coronary heart diseases or all strokes, with 14.1 million new cases and 8.2 million deaths in 2012 [1], there is a register of a continuous decline in cancer death rates that has resulted in an overall drop of 23% since 1991 [2]. Despite this progress, there is a register of 8.8 million deaths globally in 2015, and cancer is now the leading cause of death in 21 states of the United States of America [2]. The total annual economic cost of cancer in 2010 was approximately $1.16 trillion [3]. This burden is further expected to rise, with over the predicted 20 million new cancer cases expected globally by 2025 [4]. Moreover, incidence and death rates are increasing for several cancer types, for example liver and pancreas [2]. In the low- and middle-income countries, the picture is even darker, where approximately 70% of deaths are due to cancer diseases and where only one in five countries have the necessary data to drive cancer policy [3,5]. Advancing the fight against cancer requires both increased investment in cancer pathology research and in new safe, effective, inexpensive and minimal side effect anticancer agents.

For millennia, indigenous cultures around the world have used traditional herbal medicine to treat a myriad of maladies. Plants constitute a common alternative for cancer treatment in many countries, and more than 3000 plants worldwide have been reported to have anticancer properties [6,7]. Although a recent study suggests that nowadays, the traditional medicines are less used, even in populous middle-income countries [8], herbal medicine use is still common in oncology therapy worldwide [6,7,9,10,11]. In the last two decades, the use of herbal remedies has also been widely embraced in many developed countries as complementary and alternative medicine, but following tight legislation and under surveillance [12]. Natural products have garnered increasing attention in cancer chemotherapy because they are viewed as more biologically friendly and consequently more co-evolved with their target sites and less toxic to normal cells [13]. Moreover, there is evidence that natural product-derived anticancer drugs have alternative modes of promoting cell death [14,15]. Based on these facts, many researchers are now centering their investigations on the plants’ potential to deliver natural products that can become useful to the pharmaceutical industry [16,17,18]. In fact, the utilization of natural products as the background to discover and develop a drug entity is still a research hot point. From small molecules approved for cancer chemotherapy between 1940 and 2014, around 49% are natural products [19].

In spite of all the beneficial potential of medicinal plants and consequently of their products, many continue without adequate monitoring to guarantee their effectiveness and safety [20,21].

The following sections offer an overview of compounds from plants that have been found to exhibit activity against different types of cancer and are now on the market as anticancer drugs or are involved in clinic trials, which means they are involved in the last stage of the development of a clinical drug. Therefore, these compounds, which constitute successful cases in cancer therapy, will be briefly discussed.

## 2. Secondary Metabolites from Plants as Anticancer Agents

Throughout history, plants have been a rich source of affordable natural compounds, explicitly the secondary metabolites, that possess sufficient structural complexity so that their synthesis is difficult or at this time not yet accomplished and exhibit a broad spectrum of bioactivities including antitumor activity [22,23]. Secondary metabolites are mostly small organic molecules, produced by an organism, that are not essential for its growth, development and reproduction. They can be classified based on the pathway by which they are synthesized [24]. Additionally, a simple classification includes three main groups: terpenoids (polymeric isoprene derivatives and biosynthesized from acetate via the mevalonic acid pathway), phenolics (biosynthesized from shikimate pathways, containing one or more hydroxylated aromatic rings) and the extremely diverse alkaloids (non-protein nitrogen-containing compounds, biosynthesized from amino acids such as tyrosine, with a long history in medication) [24,25]. Several new cytotoxic secondary metabolites are isolated from plants each year and constitute a source of new possibilities to explore in order to fight against cancerous diseases.

Although some natural compounds have unique anticancer effects, their use in clinical practice is not possible due to their physico-chemical properties (e.g., limited bioavailability) and/or their toxicity. On the other hand, plant occurring secondary metabolites often can be excellent leads for drug development. Thus, modifying the chemical structure of these more promising compounds is one strategic way to increase their anticancer action and selectivity, improve their absorption, distribution, metabolism and excretion properties and decrease their toxicity and side effects [26,27]. Herein we will present the most significant achievements in the area of plant secondary metabolites, some already in clinical use and others in clinical trials as anticancer agents, as well as their most efficient derivatives obtained by structural modifications.

### 2.1. Metabolites Used in Cancer Therapy

During the last few decades, a wide range of cytotoxic agents was discovered from plants, but very few of these managed to reach clinical use after successfully running through the entire long, selective, expensive and bureaucratic process from their chemical identification to their effectiveness in therapeutic cancer treatment. Each of these compounds has their histories of success and limitations, which has been told by many authors and which are hereinafter counted in a historical, molecular, pharmaceutical and clinical point of view.

#### 2.1.1. Vincristine

**Figure 1:**
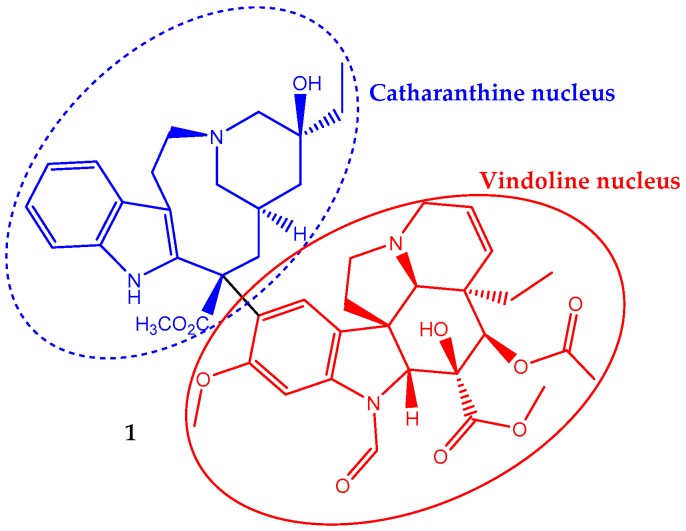
Chemical structure of the vinca alkaloid vincristine (**1**), an anticancer natural agent that repress cell growth by altering the microtubular dynamics.

Vincristine (**1**) has a non-symmetrical dimeric structure, composed of a two indole-type nucleus linked by a carbon–carbon bond, the vindoline portion and the catharanthine type portion (Figure 1). In 1963, the Food and Drug Administration (FDA) approved its clinical use to treat cancer. In fact, it was one of the first plant-derived anticancer agents approved by this agency [19]. It is a naturally-occurring alkaloid extracted from the leaves of *Catharanthus roseus* (L.) G.Don (formerly *Vinca rosea* L.) and has been used in chemotherapy in adult, but mainly in pediatric oncology practice against acute lymphoblastic leukemia. Its incorporation in the treatment regimen increases the survival rate to eighty percent [28]. It is also used to treat rhabdomyosarcoma, neuroblastoma, lymphomas and nephroblastoma [29,30].

The large interest in vincristine contrasts with its low natural occurrence, and consequently, its extraction is very expensive. This situation has stimulated an intense research effort aiming to find promising strategies to increase vincristine (and other vinca alkaloids) production. Selected enzymes’ manipulation by genetic engineering to raise the metabolic flow rate toward vincristine and the use of elicitors to activate genes involved in vincristine metabolic pathways are effective strategies to increase the biotechnological production of this compound [30,31]. However, some improvements are needed before these processes become economically viable. Another possibility to obtain more vincristine is the application/optimization of high yield extraction methodologies like negative-pressure cavitation extraction [32].

Vincristine, in a concentration-dependent manner, can affect cells’ division. However, the most well-known mechanism of vincristine antitumor activity involves interaction with tubulin, the basic constituent of mitotic spindle microtubules, inhibiting its polymerization and resulting in the suppression of mitosis. Therefore, it disrupts the assembly of the mitotic spindle, which in turn leads to the demise of actively-dividing cells [33]. Some authors report that at the lowest effective concentration, the anti-proliferative effect is due to a subtly change in the addition and loss of tubulins at the mitotic spindle microtubule and thus stabilizes the mitotic spindle assembly and disassembly processes that lead to metaphase arrest [30]. Once microtubule dynamics, and therefore cell division, can be perturbed by blocking the polymerization or depolymerization of tubulin in microtubules and thus impairing the mitotic spindle assembly, it seems that vincristine can act by both mechanisms depending on the concentration level. Moreover, a molecular docking study showed some evidence suggesting each part of the vincristine dimeric structure exhibits a specific role on its anticancer activity once the vindoline nucleus binds tubulin heterodimers, while the catharanthine nucleus provides a cytotoxic effect [34].

Despite the long history of vincristine clinical application in fighting cancer, there are three factors that diminish its impact in therapeutics: (i) its antitumor mechanism is cell-cycle-specific, and the duration of its exposure to tumor cells can significantly affect its antitumor activity; (ii) the pharmacokinetic behavior of vincristine in human blood is described by a bi-exponential elimination pattern with a very fast initial distribution half-life followed by a longer elimination half-life, and it has a large volume of distribution, suggesting diffuse distribution and tissue binding [35]; (iii) it may cause temporary or permanent peripheral neuropathy, which is a dose-dependent side effect influenced by several variables such as age, race, genetic profile and administration method, and older children, in particular Caucasian, seem to be more susceptible [36]. Some of these factors could be mitigated by encapsulation of vincristine into liposomes, which is intended to increase the circulation time, optimize delivery to target tissues and facilitate dose intensification without increasing toxicity [35].

In 2012, the FDA approved the use of sphingomyelin/cholesterol (SM/Chol) liposomal vincristine (Marqibo^®^) to treat adults with relapsed acute lymphoblastic leukemia (New Drug Application: 202497). Vincristine can be loaded into conventional liposomes like SM/Chol liposomes, but other types of liposomes, for example PEGylated liposomes, were already tested, although SM/Chol liposomal vincristine displays a relatively long circulation time, a reduced leakage rate from liposomes and an improved antitumor effect compared to PEGylated liposomal vincristine [33]. Clinical trials involving Marqibo^®^ are underway to pediatric patients with relapsed or chemotherapy-refractory solid tumors and leukemia (ClinicalTrials.gov Identifier: NCT01222780). Moreover, other vincristine encapsulated formulations are involved in clinical studies in which they are tested against other types of cancer such as small-cell lung cancer (ClinicalTrials.gov Identifier: NCT02566993), advanced cervical cancer (ClinicalTrials.gov Identifier: NCT02471027) and liver cancer (ClinicalTrials.gov Identifier: NCT00980460).

Vincristine generally exhibits better efficacy when administered in combination with other antitumor agents. In fact, combined chemotherapy can not only enhance the destruction of tumor cells, but also decrease toxicity and drug resistance with drugs exhibiting different mechanisms of action. Therefore, open clinical trials are in progress involving combined vincristine therapy (e.g., NCT02879643; NCT01527149). Very recently, a case report was done of infantile fibrosarcoma treated by adjuvant therapy after excision, using vincristine and dactinomycin, where the duration of chemotherapy was determined according to tumor response. At the end, there was no functional impairment and no evidence of recurrence at 18 months after therapy [37].

#### 2.1.2. Paclitaxel

**Figure 2:**
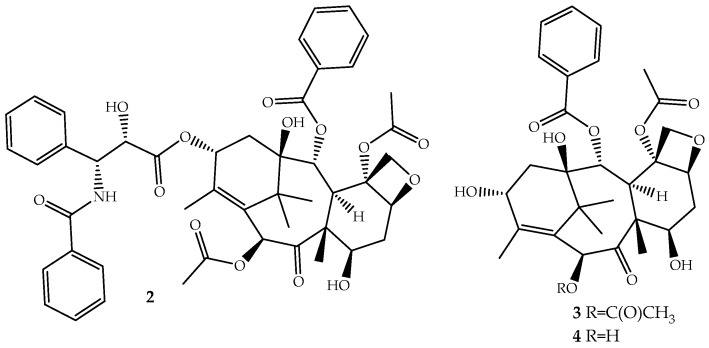
Chemical structure of paclitaxel (**2**), a natural microtubule inhibitor, and of its precursors baccatin III (**3**) and 10-deacetylbaccatin III (**4**).

The discovery of novel natural structures with significant biological relevance and with new action mechanisms have tremendous impact on the pharmaceutical industry. The discovery of (**2**) is an excellent example. Its high activity and its novel mechanism of action, tubulin-assembly promotion, is a milestone of a new era in anticancer drug discovery. Paclitaxel, isolated from the bark of *Taxus brevifolia* Nutt. (Pacific Yew) and sold under the brand name Taxol^®^ since 1993, is a complex molecule that has become one of the most active cancer chemotherapeutic drugs known [38,39]. It is a tricyclic diterpenoid, occasionally considered as a pseudo alkaloid, that contains a complex 6,8,6-tri-cycle-fused skeleton, named the “taxane” ring system, linked to a four-member oxetane ring and having alcohol, ester, ketone and amide functions (Figure 2).

Paclitaxel is a non-ionic molecule with high lipophilicity (log *P* = 3.20) that is practically insoluble in aqueous medium (aqueous solubility ~0.3–0.5 µg/mL) [40]. Due to this hydrophobicity its administration is performed in a solution containing alcohol and polyoxyethylated castor oil to enhance its delivery. The biosynthetic pathway of paclitaxel is a complex process that starts with precursor geranylgeranyl diphosphate and involves 19 steps regulated by several enzymes, and some were already characterized, but the process is not yet fully understood [41].

Although the medicinal use of paclitaxel has been achieved exclusively with purified compound from the bark of Pacific Yew, the plant’s low content and the ecological impact of its harvesting have prompted extensive searches for alternative sources. The total synthesis of paclitaxel was not successful until 1994 [42], and even after several improvements [43,44], it remains a laborious work that prevents its industrial viability. More sustainable alternatives are being used: (i) the fermentation technology with microbes or plant cell culture [45]; (ii) protein engineering to elevate catalytic fitness for paclitaxel production [46]; (iii) semisynthesis from baccatin III (**3**, Figure 2) [47] or 10-deacetylbaccatin III (**4**, Figure 2) [48], two paclitaxel precursor molecules, which are non-cytotoxic and are found in much higher quantities and readily available from the needles of *Taxus baccata*, *Taxus brevifolia* and other *Taxus* species [49]. The last approach is the one employed by the pharmaceutical industry.

The introduction of paclitaxel in the last few decades has expanded the therapeutic options, mainly due to its powerful anticancer activity, and great successes in the treatment of breast, ovarian and lung cancers have been achieved [39]. Moreover, its success is also due to effectiveness on both solid and disseminated tumors and a broad spectrum of antitumor activity predicted by its unique mechanism of action, which targets the very basic elements of the cancer phenotype like cell proliferation and DNA repair [38]. In fact, paclitaxel skeleton functional groups are at special positions and ensure that β-tubulin is targeted in order to prevent the dynamic microtubule disassembly process required for proper mitotic spindle assembly and chromosome segregation during cell division. Consequently, cell death is caused in a time- and concentration-dependent manner [38].

**Figure 3:**
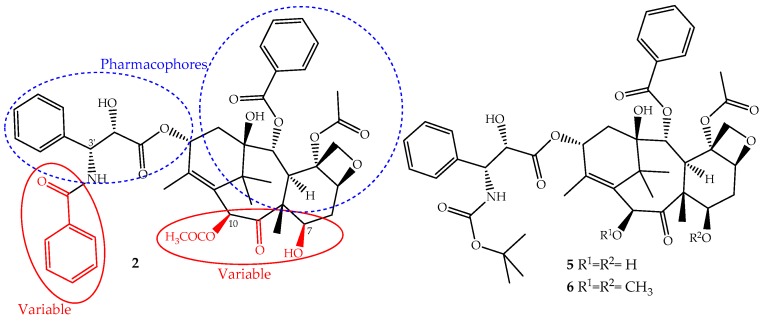
The parts of the paclitaxel (**2**) structure that could be modified without loss of activity and two of its derivatives, docetaxel (**5**) and cabazitaxel (**6**), available on the market for clinical use.

The continuous research on the mechanism of action of paclitaxel together with the structure activity relationship (SARs) and quantitative SAR (QSAR) revealed and assigned the pharmacophores, as well as structural parts that should not be modified (Figure 3). This allowed the design of novel derivatives with the best efficacy and fewer side effects [26,50]. Based on this knowledge, two semi-synthetic derivatives were developed with great success, docetaxel (**5**) and cabazitaxel (**6**) (Figure 3). They were obtained by structural modifications restricted to the variable sections of the original structure and are now available for clinical use (Figure 3).

Although paclitaxel has been applied effectively to treat many cancer diseases, its therapeutic efficacy is starting to be limited due to multidrug resistance (MDR) development [51,52]. Although the cellular mechanisms involved in the MDR are not fully understood, it appears that the overexpression of ABCB1 (also called P-glycoprotein) and ABCC10 (also named multidrug resistance protein 7) efflux transporters, the α-/β-tubulin mutations and/or alterations in the binding regions are the main cause [51,52].

The development of new drug delivery systems and new formulations allowed paclitaxel to find its way to the tumor tissue for more direct and safe anticancer activity and to overcome paclitaxel’s multidrug resistance, its poor aqueous solubility, clinical neurotoxicity and neutropenia [53,54,55]. For example, Lipusu^®^, the first paclitaxel lecithin/cholesterol liposome injectable, has been on the Chinese market since 2006 and is used in the treatment of ovarian, breast, non-SCLC, gastric and head and neck cancers [39]. This liposomal formulation Lipusu^®^ exhibited similar antitumor effects to paclitaxel, but its toxicity is lower than that of paclitaxel under the same dosage [39,56]. Another example is Abraxane^®^, an injectable nanoparticle albumin-bound paclitaxel, also named *nab*-paclitaxel developed to improve the solubility of paclitaxel, which was approved in 2005 by FDA and in 2012 by European Medicines Agency (EMA) (EMA/99258/2015, EMEA/H/C/000778) [57]. Higher doses of *nab*-paclitaxel can be administered over a shorter infusion time, and consequently, there is an improvement in neuropathy side effects after the therapy discontinuation [57], although peripheral sensory neuropathy occurred more frequently with *nab*-paclitaxel compared to paclitaxel [55].

The development of paclitaxel-mimics, with a simplified structure, also allowed the discovery of docetaxel (**5**, Figure 3), on the market since 1995 under the trade name Taxotere^®^, a drug that has fewer side effects and improved pharmaceutical properties [58]. It is obtained by semisynthesis from 10-deacetylbaccatin-III and shares with paclitaxel the same mechanism of action and identical ABCB1 affinity, but with different pharmacokinetics and side effects [49]. It is structurally different from paclitaxel only at the C-10 (acetyl group removed) and C-3′ positions (the *N*-C(O)Ph group is replaced for an *N*-*tert*-butyl acetate group), (Figure 3) alterations that increase its water solubility and lower its lipophilicity (log *P* = 3.20). It belongs to the first generation of taxanes, used for the treatment of breast, ovarian, prostate and non-SCLCs, and exhibits a longer half-life, more rapid cellular uptake and longer intracellular retention than paclitaxel [59].

Cabazitaxel (Jevtana^®^) (**6**, Figure 3) was approved by the FDA in 2010 for the treatment of patients with hormone-refractory metastatic prostate cancer and tumors that are docetaxel- or paclitaxel-resistant [60]. It is also obtained by semisynthesis and is a dimethoxyl derivative of docetaxel, a structural change that increases its lipophilicity (log *P* = 3.90) and consequently its cell penetration through passive influx associated with alteration of the P-gp affinity [61]. This allows the drug to accumulate intracellularly at greater concentrations than docetaxel and explains its improved cytotoxicity and effectiveness in taxane-resistant patients [27,49].

Paclitaxel is already a blockbuster of the pharmacy industry not only due to the development of new delivery systems in cancer therapy [62] and its application in combination with other anticancer drugs (e.g., ClinicalTrials.gov Identifier: NCT02379416, NCT00584857 and NCT01288261) [63,64], but also due to its use in clinical trials for other treatments such as psoriasis [65] and botulinum neurotoxin inhibiting [66], just to mention a few examples that ensure this compound’s success.

#### 2.1.3. Homoharringtonine

**Figure 4:**
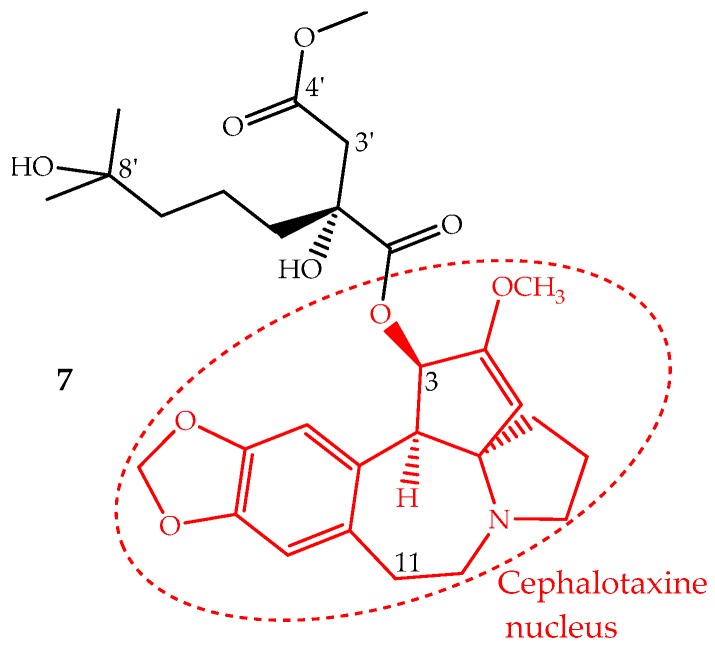
Chemical structure of homoharringtonine also named omacetaxine mepesuccinate (**7**) with alkaloid nucleus cephalotaxine (red).

Homoharringtonine (**7**) is an alkaloid with a cephalotaxine nucleus named cephalotaxine 4-methyl-2(*R*)-hydroxy-2-(4-hydroxy-4-methylpentyl)succinate (Figure 4). It was first isolated from *Cephalotaxus harringtonii* (Knight ex J.Forbes) K.Koch and *Cephalotaxus fortunei* Hook. trees, whose bark extracts were used in Chinese traditional medicine to treat cancer. Homoharringtonine and other cephalotaxine derivatives can also be found in leaves, bark and seeds of other *Cephalotaxus* species [67]. In fact, the cephalotaxine itself is very abundant in *Cephalotaxus* species leaves which can be isolated and transformed by a simple esterification into homoharringtonine, and thus, this procedure constitutes a semisynthetic methodology used for homoharringtonine industrial production [50,68].

The interest in homoharringtonine started when its potent antiproliferative activity against murine P-388 leukemia cells with IC_50_ values of 17 nM was demonstrated [69]. In fact, since the 1970s homoharringtonine or a mixture of cephalotaxine esters has been used in China to treat hematological malignancies [70]. However, only after the development of the above-mentioned semisynthetic procedure did homoharringtonine attract the attention of Western medicine.

Homoharringtonine is a first-in-class protein translation inhibitor, which means that it inhibits the elongation step of protein synthesis. In fact, homoharringtonine binds to the A-site of the large ribosomal subunit, an action that blocks the access of the charged tRNA and consequently the peptide bond formation [71]. Since this drug does not target specific proteins, its success is mainly due to the fact that it can disturb proteins with rapid turnover such as the leukemic cells’ upregulated short-lived oncoproteins BCR-ABL1 and antiapoptotic proteins (Mcl-1, Myc) leading to cells apoptosis [71]. Recently, other mechanisms indicated that it could also affect signaling pathways, like the Jak-stat5 pathway, by regulating protein tyrosine kinase phosphorylation [72] and by activating the TGF-β pathway through phosphorylation of smad3 [73].

The identification of several natural cephalotaxine esters structurally similar to homoharringtonine and other derivatives obtained by semisynthesis allowed establishing some structure activity relationships, which were recently reviewed and discussed by Chang et al. [69]. The most important SAR are: (i) the cephalotaxine nucleus is much less active against the P388 cell line than its esters derivatives; (ii) an aliphatic side chain bonded to the hydroxyl group at C-3 seems to be necessary to enhance the activity; (iii) the presence of hydroxyl groups at C-11 or C-3′ decreases the activity; (iv) a free carboxylic acid at C-4′ abruptly decreases the activity; however, the methyl group can be replaced by other alkyl groups, even bulky ones, without the loss of the activity and in some cases enhancing it; (v) bulky groups bonded to 8′-OH are also tolerated; (vi) substituents bonded at 2′-OH imply a significant loss of activity (Figure 4).

There is a long track record of the clinical efficacy and safety of homoharringtonine use in the treatment of chronic myeloid leukemia. Currently, the focus is on its use in patients that experienced resistance or intolerance to multiple tyrosine kinase inhibitors (sorafenib and imatinib target) [74] and in patients carrying the T315I mutation, a variant that is unresponsive to tyrosine kinase inhibitors [74,75,76]. In fact, homoharringtonine was approved by the FDA in 2012 (sold under the trade name Synribo^®^) to be used in the treatment of chronic myeloid leukemia in patients with resistance and/or intolerance to two or more tyrosine kinase inhibitors, and it is the only natural therapeutic agent approved as a commercial drug to treat chronic myeloid leukemia.

The commercial approval of homoharringtonine and continued preclinical and clinical investigations of this compound indicate opportunities for its use in other hematological malignancies. For instance, the produced durable hematologic and cytogenetic responses regardless of mutational status [76,77] exhibit the ability to effectively kill stem/progenitor cells [77,78] and have a role in acute myeloid leukemia [79].

The homoharringtonine therapeutic efficiency continues to be evaluated, and its use is expect in the near future in other hematologic malignancies. It is being evaluated in 20 clinical trials, in mono and combined therapy, involving, for example, patients with newly-diagnosed acute myelogenous leukemia (NCT01873495), with relapsed/refractory acute myeloid leukemia carrying FLT3-ITD (NCT03170895), with myelodysplastic syndrome (NCT02159872), and in combined therapy with imatinib mesylate (NCT00114959), with quizartinib (NCT03135054) and with cytarabine and idarubicin (NCT02440568). Moreover, the subcutaneous administration of homoharringtonine does not influence its bioavailability (NCT00675350) [80] and allowed decreasing its cardiac toxicity [77]. Additionally, the FDA in 2014 approved its administration at home by the patient or a caregiver, which is indeed an improvement because patients have the opportunity to self-administer their therapy and due to homoharringtonine’s stability [81].

Although homoharringtonine treatment may result in some hematologic toxicity such as myelosuppression, this should not prevent the use of this drug, once the benefits exceed the damage and the latter can be limited mainly by adequate dose adjustment and patient training for symptoms [82]. All these data show a large number of scenarios where homoharringtonine use is applied and suggest many others where it can receive approval in the near future, showing that its long history in cancer therapy is far from over.

### 2.2. Metabolites in Clinical Trials

In September 2007, a total of 91 plant-derived compounds was in clinical trials [83], whereas at the end of 2013, there were 100 unaltered natural products plus their derivatives involved in clinical trials, with a majority being in oncology [68].

Several semisynthetic derivatives from the plant-derived compounds camptothecin (e.g., gimatecan), combretastatin A (e.g., fosbretabulin tromethamine; combretastatin A1 diphosphate), rohitukine (e.g., alvocidib, riviciclib), triptolide (e.g., minnelide) and daidzein (e.g., phenoxodiol) [50,68,83] are in clinical trials, while the lead compounds are not involved in any clinical studies as an anticancer agent, although they exhibit relevant cytotoxic properties. Only the plant-derived lead compounds are presently in clinical trials as anticancer agents, and their derivatives are discussed below.

#### 2.2.1. Ingenol Mebutate

**Figure 5:**
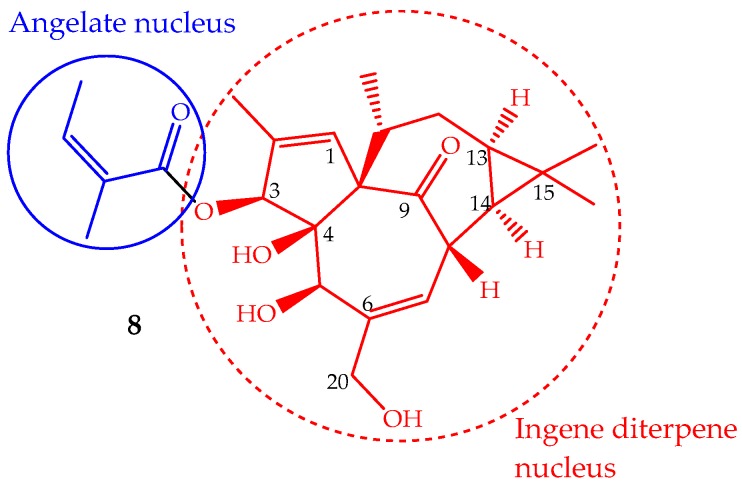
Chemical structure of the diterpene ingenol mebutate (**8**).

The phytochemical study of *Euphorbia peplus* L. latex sap yielded several macrocyclic diterpenes [84], including ingenol mebutate (**8**, Figure 5) (also known as PEP005, ingenol-3-angelate and 3-ingenyl angelate), which was later on identified as the most active antitumor component [85]. In fact, the *Euphorbia peplus* sap has been shown, in a recent phase I/II clinical study, to be effective against human non-melanoma skin cancer [86]. This ingenene-type diterpene (Figure 5) can also be isolated from other *Euphorbia* species such as *Euphorbia paralias* L., *Euphorbia millii* Des Moul., *Euphorbia palustris* L., *Euphorbia marginata* Pursh and *Euphorbia helioscopia* L., and especially in the lower leafless stems of *Euphorbia myrsinites* L., where it is found in high quantity (547 mg/kg of dry weight) [68,87]. Ingenol mebutate has been prepared by semisynthesis using ingenol, which is isolated from the seeds of *Euphorbia lathyris* L. (yield ∼100 mg/kg). The methodology involves a selective esterification of the hydroxyl group at position 3 with (*Z*)-2-methylbut-2-enoic acid (angelate nucleus) (Figure 5) [88]. Some efforts have been made to accomplish the ingenol total synthesis, but they are not suitable for application in the pharmaceutical industry, so the ingenol mebutate total synthesis remains undone. Ingenol mebutate is a monoester considered, in pharmacological terms, a small molecule. Its stability is pH dependent and can undergo facile acyl migration involving the hydroxyl groups, mainly the 5- and 20-OH (Figure 5). This characteristic is important from the biological activity point of view, because the free hydroxyl groups and the ester moiety at position 3 are required for the anticancer activity [89].

Ingenol mebutate showed potent antiproliferative effects in a dose- and time-dependent manner against several cell lines [90,91], especially against colon 205 cells line with IC_50_ = 10 nM, that means more active than staurosporine (IC_50_ = 29 nM) or doxorubicin (IC_50_ = 1.5 µM), known active compounds used as standards [90]. There is evidence that its effectiveness at damaging the tumor vasculature is related to the fact that it can be transported through the epidermis into the deep dermis via a P-glycoprotein [92]. Treatment with this compound, both in vitro (230 µM) and in vivo (42 nmol), rapidly caused swelling of mitochondria probably by loss of mitochondrial membrane potential and cell death by primary necrosis and is, therefore, unlikely to have its activity compromised by the development of apoptosis resistance in tumor cells [86]. There is evidence that this rapid action of ingenol mebutate is due to its dual action combining cytotoxic and immunomodulatory effects in which rapid lesion necrosis and antibody-dependent cellular cytotoxicity mediated by neutrophils occur [93]. The mechanism of action of ingenol mebutate is also partially related to the modulation of protein kinase C (PKC) to which it has a potent binding affinity by activating PKCδ and inhibiting PKCα [91,94]. In an in vitro assay, low isozyme selectivity was verified with a Ki ranging from 0.105–0.376 nM [95].

The above-mentioned results support the potential of ingenol mebutate for further improvements in cancer therapy; in fact, the cutaneous treatment of non-hyperkeratotic, non-hypertrophic actinic keratosis (a precancerous condition, that if untreated usually leads to a melanoma) with a gel formulation of ingenol mebutate (formerly PEP005 and marketed as Picato^®^) was approved by both FDA and EMA agencies in 2012 [96,97]. Unfortunately, adverse reactions associated with this application have been reported, although they are restricted to moderate “local skin responses” and included erythema, flaking/scaling, swelling, crusting, erosion/ulceration and vesiculation/postulation. However, it shows a favorable safety and tolerability profile exhibiting a lack of systemic absorption and photosensitivity [92,97].

#### 2.2.2. Curcumin

**Figure 6:**
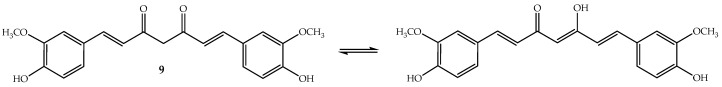
Chemical structure of the polyphenol curcumin (**9**).

Curcumin (**9**, Figure 6) or diferuloylmethane (bis-α,β-unsaturated β-diketone) is a polyphenolic compound that has been extracted from the rhizome of turmeric (*Curcuma longa* L.), a tropical Southeast Asia plant mainly used as a spice. However, the turmeric powder, which has 2–5% of curcumin, is used in Chinese and Indian traditional medicines [98]. To this ancient remedy have been attributed a wide range of beneficial properties including anti-inflammatory, antioxidant, chemopreventive, chemotherapeutic and chemo-sensitizing activity [98]. Curcumin is an orange-yellow crystalline lipophilic phenolic substance that, in solution, exists in equilibrium with its keto-enol tautomeric forms (Figure 6). It is not very soluble in water and also not very stable, although its degradation increases in basic medium [99].

Research interest in curcumin’s anticancer properties has been developed based on the low rate occurrence of gastrointestinal mucosal cancers in Southeast Asian populations and its association with regular turmeric use in their diet [100].

A large volume of experimental data established the therapeutic efficacy of curcumin in in vitro cellular level, as well as in some ex vivo tumor-derived cancer cells/solid tumors like brain tumors, pancreatic, lung, breast, leukemia, prostate, skin cancers and hepatocellular carcinoma, including cytotoxic effects on cancer stem cells and antimetastatic activity [101,102,103]. This year, its possible application in colorectal, head and neck cancer chemotherapy was also reviewed [104,105]. Equally important were the assays demonstrating that curcumin was not cytotoxic to normal cells at the dosages required for therapeutic efficacy against the cancer cell lines [106,107]. The scientific interest and pharmacological potential of curcumin anticancer effects becomes also evident from the number of patents on curcumin-based therapeutics registered in the last five years [108].

Several studies have shown that curcumin can modulate a variety of cancer-related targets or pathways [102,103,109,110], which may be responsible for its effectiveness in combating cancer diseases. Recent studies demonstrate that curcumin’s mechanism of action includes: (i) modulation of CYP enzymes by elevation of transcription factor Nrf2 level via the mitogen-activated protein kinase (MAPK) signaling pathway and Akt pathway [111]; (ii) mitotic catastrophe induction due to caspase activation and mitochondrial membrane polarization [14]; (iii) promotion of autophagic cell death, an important death inducer in apoptosis resistant cancer cells by beclin-1-dependent and independent pathways [14,112]; (iv) arrest of the cell cycle at the check points G1, S-phase and G2/M phase, modulating the cell cycle regulators, including upregulation of cyclin-dependent kinase inhibitors (CDKIs) [113]; (v) promotion of the inhibition of transcription factor NF-κB by preventing nuclear translocation of NF-κB and attenuating the DNA binding ability of NF-κB, contouring the problem of chemoresistance [114]; (vi) promotion of the inhibition of the crucial steps to angiogenesis by downregulation of the PGDF, VEGF and FGF expression and downregulation of MMPs via NF-κB, ERKs, MAPKs, PKC and PI3K inhibition [115]; and (vii) inhibiting tubulin polymerization, that is curcumin binds with DNA [116,117]. Despite this knowledge about curcumin’s multiple mechanisms of action, its biological properties are not fully understood. For example, does curcumin’s survival and proliferative effects depend on its concentration, treatment period and cells type? On the other hand, administered doses of curcumin have been studied. Systematic in vivo doses up to 300 mg–3.5 g/kg b.w. (administered for up to 14–90 days) or clinical studies with oral intake of 1.2–12 g daily (for 6 weeks–4 months) did not demonstrate any adverse effects at the populations, animals and patients [118], although these values exceed that normally consumed (granted an acceptable daily intake level of 0.1–3 mg/kg b.w. by the Joint FAO/WHO Expert Committee on Food Additives) and also the typical intake of the Indian population (60–100 mg per day).

Moreover, curcumin has been reported to act as a chemosensitizer for some clinical anticancer drugs (e.g., gemcitabine, paclitaxel and 5-fluorouracil, doxorubicin) and exhibits a synergistic effect in combination with other natural products (e.g., resveratrol, honokiol, epigallocatechin-3-gallate, licochalcone and omega-3), aspects that could be used as an effective strategy to overcome tumor resistance and reduce recurrence [108,119,120]. These observations therefore suggest that a superior therapeutic index may be achieved with curcumin when used in combination and could be advantageous in the treatment of some tumors. Anyway, additional studies are still needed to assess the exact mechanism of curcumin’s synergic effect.

Nevertheless, the clinical translation of curcumin has been significantly hampered since it is poorly absorbed, improperly metabolized and shows poor systemic bioavailability, which mandates that patients consume up to 8–10 grams of free curcumin orally each day, in order for detectable levels in the circulation [109,118]. Thus, several strategies have been proposed to counter the bioavailability issue of curcumin involving (i) the use of adjuvants like piperine, which interferes with curcumin metabolism by glucuronidation, (ii) curcumin formulations based on nanotechnology with liposomes, micelles, phospholipid, among others, and (iii) use of curcumin analogues [117,121,122,123]. As result of the anticancer potential of curcumin and despite its clinical therapeutic limitations, there are currently 17 open clinical studies involving curcumin, mainly studies of combined curcumin therapy with other substances for the treatment of several types of cancer.

#### 2.2.3. Betulinic Acid

**Figure 7:**
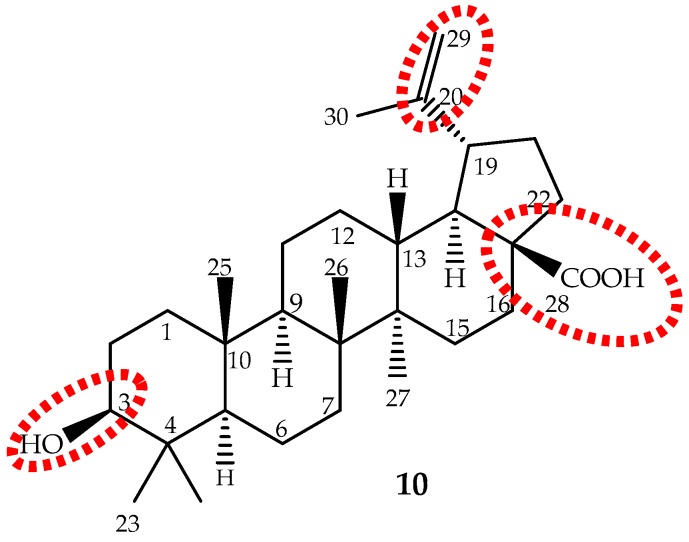
The chemical structure of the pentacyclic triterpene betulinic acid (**10**) and the main target to structural modifications (red).

Betulinic acid (3β-hydroxy-lup-20(29)-en-28-oic acid), a lupane-type pentacyclic triterpene (**10**, Figure 7) is biosynthesized from six different isoprene units and was first identified and isolated from *Gratiola officinalis* L. and named “graciolon”. It was also isolated from other species, but identified with different names (from the bark of *Platanus acerifolia* (Aiton) Willd. named “platanolic acid” and from *Cornus florida* L. named “cornolic acid”), which led to some confusion. Later on, it was confirmed that all have the same structure, and the compound was named betulinic acid. Nowadays, it is known that this triterpene is extensively spread throughout the plant kingdom (for instance *Betula* spp., *Diospyros* spp., *Syzygium* spp., *Ziziphus* spp., *Paeonia* spp., *Sarracenia flava* L., *Anemone raddeana* Regel and *Lycopodium cernuum* L., among others) and in considerable amounts (up to 2.5%) [124]. However, these sources are not sufficient to meet the growing demand for betulinic acid. Therefore, it can be obtained through a selective oxidation of betulin (lup-20(29)-en-3,28-diol) [125], far more abundant (up to 30%) in birch bark than betulinic acid [126].

In 1995, the first betulinic acid antitumor activity was reported by a researcher at the University of Illinois. It killed melanoma cells in mice with low IC_50_ values (IC_50_ 0.5–1.5 μg/mL) [127]. Since then, a number of researchers have conducted laboratory tests on betulinic acid to determine its antitumor properties, especially with respect to melanoma cells [128]. More recent studies suggest that betulinic acid possesses a broader spectrum of activity against other cancer cells, and consequently betulinic acid has been selected by the National Cancer Institute for addition into the Rapid Access to Intervention in Development (RAID) program.

Betulinic acid exhibits significant in vitro cytotoxicity in a variety of tumor cell lines and also inhibits the growth of solid tumors in vivo, comparable to some clinically-used drugs and showing a good selectivity index for cancer over normal cells even at doses up to 500 mg/kg b.w. [14,127,129,130]. Its anticancer proprieties have been demonstrated against colorectal lung, colon, breast, prostate, hepatocellular, bladder, head and neck, stomach, pancreatic, ovarian and cervical carcinoma, glioblastoma, chronic myeloid leukemia cells and human melanoma with IC_50_ values mainly between 1 to 13.0 μg/mL [14,124,128,129,130,131,132].

Betulinic acid exhibits potent anticancer activity by multiple molecular targets, the best characterized mechanism being the induction of apoptosis by direct regulation of the mitochondrial apoptotic pathway; which can be associated with mitochondrial collapse through direct opening of the permeability transition pore, decreasing mitochondrial outer membrane potential, downregulation of Bcl-2 family members, release of pro-apoptotic factors such as cytochrome c, increase of caspase activities, attenuating both constitutive and inducible STAT3 phosphorylation, nuclear translocation and its DNA binding [124,130,133]. However, there is also evidence that, in some cases, apoptosis may be induced by stabilizing p53 and downregulating NF-kB-mediated signaling [124,134].

The antimetastatic effect of betulinic acid seems to be through the prevention of the epithelial-to-mesenchymal transition in highly aggressive melanoma cells [131], while in breast cancer cells, it be by downregulation of the matrix metalloproteinases expression [133]. Betulinic acid can also induce an antiangiogenic response under hypoxia mediated by the STAT3/HIF-1α/VEGF signaling pathway [124,130], can block the cell cycle in the G1 phase through inhibition of Cyclin B1 and Hiwi in mRNA and potently induces autophagy as a survival mechanism in response to permeability transition pore opening and mitochondrial damage [14,133]. Recently, a new cell death pathway was attributed to betulinic acid in which cell death is induced through the inhibition of the stearoyl-CoA-desaturase (SCD-1), an enzyme that is overexpressed in tumor cells [135]. Proteasome inhibition assays suggest the proteasome is the main target for betulinic acid [136]. However, the regulatory effects of betulinic acid on the NF-κB pathway and on Bax or Bak expression are not well clarified [130].

Betulinic acid seems to be a very effective chemosensitizer for anticancer drug treatment in chemoresistant cell lines once it promotes the inhibition of multidrug resistance proteins in vivo and in vitro, as for example in combination with 5-fluorouracil (5-FU) and oxaliplatin [133,137]. These results clearly demonstrate that in some cases, it is possible to circumvent acquired chemoresistance by combination therapy of anticancer drugs with chemosensitizers as betulinic acid. Moreover, betulinic acid has strong synergy with mithramycin A on the inhibition of migration and invasion of pancreatic cancer cells at nontoxic concentrations by suppressing the Sp1 and uPAR level [138]. Furthermore, a synergistic effect of betulinic acid and tumor necrosis factor-related apoptosis-inducing ligand (TRAIL) combination to inhibit liver cancer progression in vitro and in vivo through targeting the p53 signaling pathway [139] revealed that betulinic acid combined with TRAIL has potential value against liver cancer.

Betulinic acid is slightly soluble in water, and therefore, its water solubility is a drawback that should be overcome to improve its absorption and bioavailability. The main targets for structure activity studies were the C-3 hydroxyl, C-20 vinyl and C-28 carboxyl groups (Figure 7). The 3-OH oxidation increased cytotoxic activity, but decreased selectivity; introduction of groups, such as amine or hydroxyl, at the C-28 position increased activity; while modifications at C-20 did not enhance cytotoxicity [14,124,140]. It can be conclude that modifications may improve the cytotoxicity and/or the water solubility, but not the selectivity. It seems that the presence of the free hydroxyl group at C-3 and the carboxyl group at C-28 are the most important features.

Recently, a clearer and more realistic method, 3D-QSAR by CoMFA and CoMSIA, shows the structure-cytotoxicity relationship of betulinic acid derivatives against human ovarian cancer cell A2780, and the main conclusions were: an electropositive group at the C-2 α-site; an electronegative and hydrogen bond acceptor group at the C-2 β-site; bulky groups at the C-3 β-site; bulky and electronegative groups at the C-3 α-site; bulky, electronegative and hydrogen bond donor or acceptor groups at the C-28 side chain; and would be beneficial to the antitumor potency (Figure 7) [130].

Due to its extraordinary potential as an antitumor agent, betulinic acid was involved in phase I/II clinical trials to evaluate its safety and effectiveness. The study involved topical applications (20% betulinic acid in ointment) to treat dysplastic nevi that can transform into melanoma. Unfortunately, at the end of 2013, the study was suspended due to funding issues (Clinical Trials database).

## 3. Conclusions

Cancer is becoming a high profile disease in developed and developing countries, and its treatment is a struggle with some successful cases. Nevertheless, the drugs developed by synthesis and used in chemotherapy have limitations mainly due to their toxic effects on non-targeted tissues and consequently furthering human health problems. Therefore, there is a demand for alternative treatments, and the naturally-derived anticancer agents are regarded as the best choice. As demonstrated herein, with some representative examples, secondary metabolites are themselves suitable anticancer agents leading to the development of new clinical drugs with also new anticancer mechanisms of action. Some have already become cases of success for the pharmaceutical industry. Additionally, they are excellent lead compounds, by which, through structural modifications, alternative formulations and/or using increasingly effective delivery systems, their pharmacological potential is enhanced. Recent new biotechnological solutions, using nanotech approaches, present a new hope for cancer therapy (e.g., plant drug-functionalized nanodiamonds and other nanocarriers based on anticancer drugs). Simultaneously, they provide a further step forward in the successful use of secondary metabolites for cancer therapeutic purposes [141,142,143,144]. In other cases, the success story has not yet reached its high point with its introduction in the market, but the more recent studies presented and discussed in this paper clearly show that this goal is getting closer. On the other hand, demand for plant-derived drugs is putting pressure on high-value medicinal plants and risking their biodiversity, so exploitation of these agents needs to be managed to keep up with demands and be sustainable. Fortunately, there are currently developments using new biotechnological solutions and sustainable alternative methods for the production of high-value plant metabolites.

## Abbreviations

5-FU	5-fluorouracil
A2780	human ovarian carcinoma cell line
ABCB1	ATP binding cassette subfamily B member 1
ABCC10	ATP binding cassette subfamily C member 10
Akt	serine/threonine-specific protein kinase
b.w.	body weight
Bak	pro-apoptotic Bcl-2 protein
Bax	bcl-2-like protein 4
Bcl-2	B-cell lymphoma 2 protein
BCR-ABL1	breakpoint cluster region protein-Abelson murine leukemia viral oncogene homolog 1
CDKI	cyclin-dependent kinase inhibitors
colon 205	human Caucasian colon adenocarcinoma cell line
CoMFA	comparative molecular field analysis
CoMSIA	comparative molecular similarity index analysis
CYP	cytochrome P450
DNA	deoxyribonucleic acid
EMA	European Medicines Agency
ERK	extracellular signal-regulated kinases
FAO	Food And Agriculture Organization
FDA	Food And Drug Administration
FGF	fibroblast growth factor
FLT3-ITD	fms-related tyrosine kinase 3 internal tandem duplication
HIF-1α	hypoxia-inducible factor 1-alpha
HMGB1	high mobility group box 1 protein
IC_50_	half maximal inhibitory concentration
MAPK	mitogen-activated protein kinase
Mcl-1	induced myeloid leukemia cell differentiation protein
MDR	multidrug resistance
miRNA	micro-ribonucleic acid
MMP	matrix metalloproteinase
mRNA	messenger ribonucleic acid
Myc	proto-oncogene
*nab*-paclitaxel	nanoparticle albumin-bound paclitaxel
NF-κB	nuclear factor kappa B cells
Nrf2	nuclear factor (erythroid-derived 2)-like 2
P-388	bipotential murine pre-B cell lymphoma
PEP005	ingenol mebutate
PGDF	platelet-derived growth factor
P-gp	p-glycoprotein
PI3K	phosphatidylinositol-4,5-bisphosphate 3-kinase
PICN	paclitaxel injection concentrate for nanodispersion
PKC	protein kinase C
PKCα	protein kinase C-α
PKCδ	protein kinase C-δ
QSAR	quantitative structure activity relationship
RAID	rapid access to intervention in development
SAR	structure activity relationship
SCD-1	stearoyl-CoA- desaturase 1
SCLC	small-cell lung cancer
SIRT1	NAD-dependent protein deacetylase sirtuin-1
SM/Chol	sphingomyelin/cholesterol
smad3	mothers against decapentaplegic homolog 3
Sp1	specificity protein 1
STAT3	signal transducer and activator of transcription 3
T315I	mutation resulting in an amino acid substitution at position 315 in BCR-ABL1, from a threonine (T) to an isoleucine (I).
TGF-β	transforming growth factor beta
TRAIL	tumor necrosis factor-related apoptosis-inducing ligand
tRNA	transfer ribonucleic acid
uPAR	urokinase receptor
VEGF	vascular endothelial growth factor
WHO	World Health Organization
